# Talk CPR - a technology project to improve communication in do not attempt cardiopulmonary resuscitation decisions in palliative illness

**DOI:** 10.1186/s12904-018-0370-9

**Published:** 2018-10-19

**Authors:** Mark Taubert, James Norris, Sioned Edwards, Veronica Snow, Ilora Gillian Finlay

**Affiliations:** 1 Palliative Care Department, Velindre University NHS Trust, Cardiff, CF14 2TL UK; 2 Digital Legacy Association, London, UK; 3 School of Medicine, Cardiff University, Cardiff, UK; 4 Byw Nawr Coalition/Dying Matters in Wales and NHS End Of Life Care Board Wales, Cardiff, UK; 5 Palliative Care Department, Velindre University NHS Trust, Cardiff, UK

**Keywords:** Resuscitation, Not for resuscitation, DNR, DNACPR, Advance care planning, Web resources, Video evaluation

## Abstract

**Background:**

A national Do Not Attempt Cardiopulmonary Resuscitation policy was rolled out for the National Health Service in Wales in 2015. A national steering group led on producing information videos and a website for patients, carers and healthcare professionals, forming part of a quality improvement program. Videos were planned, scripted and produced with healthcare professionals and patient/carer representatives, and were completed with both English and Welsh language versions. The TalkCPR videos encourage and promote open discussion about Cardiopulmonary Resuscitation (CPR) and DNACPR in palliative care situations.

**Methods:**

We worked with patient/carer groups to evaluate whether video resources to convey the salient facts involved in CPR and DNACPR decisions for people with palliative and life-limiting illness were acceptable or not. We conducted a mixed-method design service review in five phases to evaluate whether this technological resource could help. After creating video and website materials, they were evaluated by doctors, nurses and a patient/carer group. We also sent out one lightweight TalkCPR video media pad to each practice in Wales. These rechargeable electronic video media pads had communication videos pre-loaded for easy viewing, especially in areas with poor roaming data coverage.

**Results:**

Videos were demonstrably acceptable to both patient and carer groups, and improved healthcare professional confidence and understanding. Videos went live on the TalkCPR website, in all Welsh Health Boards and on Youtube, and are now used in routine practice throughout Wales.

**Conclusion:**

This is the first time that DNACPR information videos are aimed directly at palliative care patients and carers, to explore this sensitive subject with them, and to encourage them to approach their doctor or nurse about it. The website, app and video media pads were developed by patients, the Digital Legacy Association, Welsh NHS IT services, Welsh Government, the Bevan Commission and the Dying Matters Charity in Wales ‘Byw Nawr’. The GMC, the Royal College of General Practitioners and NICE have listed TalkCPR as a learning resource. There has also been a collaboration with Falmouth University Art College, who helped produce graphic designs to facilitate and encourage discussions about CPR and end of life care.

**Electronic supplementary material:**

The online version of this article (10.1186/s12904-018-0370-9) contains supplementary material, which is available to authorized users.

## Background

How can a palliative patient express his or her views on future emergency care options, if they have little or no information about what procedures like CPR (Cardiopulmonary Resuscitation) involve, beyond what they have seen on television programmes and in fictional media? One of the authors of this research study, MT, outlined these challenges in a Guardian newspaper article, in particular how this area of medicine is often misrepresented on television [1].

The resuscitation council has stressed the importance of discussing DNACPR (Do Not Attempt Cardiopulmonary Resuscitation) decisions with patients and proxy, and two recent high profile court cases have changed practice [2]. Not discussing DNACPR, when a form has been added to medical notes can be unlawful. The National Centre for Health and Care Excellence (NICE) has set out standards for end of life care stressing early, anticipatory planning [3]. Fritz has talked about a ‘duty to consult’ [4]^.^

Face-to-face discussions with a healthcare professional are important, but additional media resources can and should be used to help understand DNACPR towards the end of life and in palliative care settings [5]. Such resources may prompt further, more informed discussions between healthcare professionals and patients/proxy. Patient information leaflets are increasingly relied upon in nearly all areas of medicine [6], but our patient groups have told us that they are often not read. The national DNACPR steering group in Wales set out to bridge this information gap, and video resources were considered to be a useful means to provide information. Four videos were planned, two aimed at palliative care patients and the general public, and two further videos for healthcare professionals.

Cardiopulmonary Resuscitation or CPR is an emergency procedure, with the aim of restarting heart and breathing when these have ceased to function [7]. This is a medical emergency, and CPR can be successful in particular in the young and in those where a defibrillator can treat a reversible fatal rhythm. Increasingly, CPR has been given in situations when it is either very unlikely to work, or is not going to work at all, for instance in slow deterioration towards the end of life in patients with chronic, long term conditions such as cancer and/or when people are in the last moments of life, dying naturally. Hospital cardiac arrest teams and ambulance crews in the community have a duty to treat on arrival at a scene, and do not always know the prior health status of an individual they are called urgently to see. Success rates of CPR in people with cancer that has spread, are as low as 1.9% [8].

Witnessing and undergoing CPR can be traumatic and involves chest compressions, intravenous lines being placed, insertion of airways, electric shocks to the bare chest. Rib fractures, internal bleeding, severe pain, collapsed lungs and brain damage can be short and longer term problems, if the patient survives.

Talking about CPR is important, and in fact it is best to discuss it when a person is still reasonably well, and can express an opinion on whether the intervention is something they would consider appropriate for themselves or not, should it become necessary in future. There is a need to explain this procedure better within society, and also to create reproducible ways of giving clinicians opportunities to gain confidence in talking about this challenging topic more.

## Methods

We conducted a mixed-method design service review in five phases to evaluate whether this technological resource could help. These five implementation phases are described below.

### PHASE 1- patient/carer engagement event

**Fig. 1:**
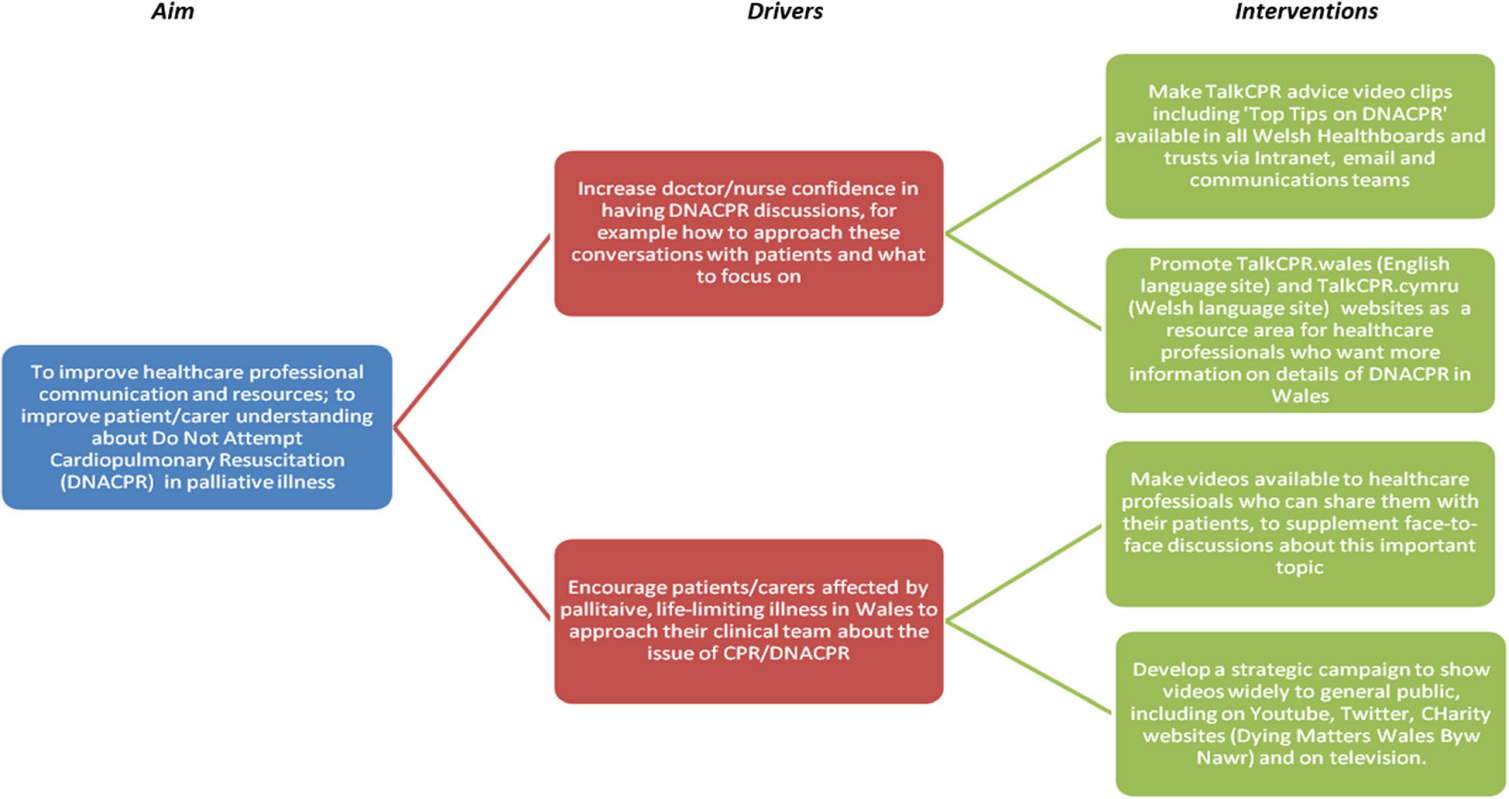
Driver diagram that helped set out strategic aim for TalkCPR project in Wales

At the start of the project, Velindre NHS Trust’s patient/carer group were asked to comment on whether they considered videos, as a medium, to be appropriate for the purpose of conveying the key areas for this topic. The Patient/Carer Liaison Group (PLG) consisted at the time of enquiry of 24 individuals with either a background as a cancer patient, or as a current or past carer of someone with cancer. The hospital trust also has a patient and carer information and support co-ordinator, who was instrumental in setting up this meeting. Patients and carers unanimously agreed that these videos would be important and of high value and were very supportive of the TalkCPR project. Patient/carer representatives in our hospital felt it was important that healthcare professionals approach this topic *not* as a formulaic process, but that they respect this as one of the most important conversations the patient and carers may have in their disease journey. They also felt that conveying a message that other measures, like antibiotics, chemotherapy, blood products, emergency fluids would *not* be left unconsidered, merely because of the presence of a DNACPR form. It was felt important by the group to convey this and that it would provide reassurance to patients. A Driver Diagram to further refine these aims and interventions was created (Fig. 1).

### PHASE 2- baseline exercise and aspirations

In response to the aims collated and set out in Phase 1, the TalkCPR Implementation group, consisting of clinicians, patients, nursing staff, resus officers and a psychologist, evaluated the patient/carer suggestions and noted key aspirations for the TalkCPR project. The main author, Dr. Mark Taubert suggested the campaign name “Talk CPR” and the Twitter hashtag #TalkCPR and this name was unanimously agreed upon. An additional meeting with the chair of the patient group and a film production team was held, and also included a psychologist, a resuscitation officer, an oncologist, a palliative care nurse and the chair of the national Do Not Attempt Cardiopulmonary Resuscitation steering group.

The key aim that was agreed on, as part of this baseline review, was outlined as follows:

- TalkCPR aims to improve healthcare professional communication and resources; and to improve patient/carer understanding about Do Not Attempt Cardiopulmonary Resuscitation (DNACPR) in palliative illness

Two key drivers emerged during this baseline exercise. Patients and healthcare practitioners felt it was important to:

- Increase doctor/nurse confidence in having DNACPR discussions, for example how to approach these conversations with patients and what to focus on.
- Encourage patients/carers affected by palliative, life-limiting illness in Wales to approach their clinical team about the issue of CPR/DNACPR.

All meetings were minuted and consensus was sought on what should emerge as content for the videos. The question: “What should the goal of this video information project be?” was asked at the start of the meetings, and all participants were asked to give their views. Aspirations were written down and then altered by participants until everyone was content that they represented what they considered most informative. From this, four aspirational interventions were agreed upon by the patients and healthcare professionals on the TAlkCPR group:

- DNACPR advice videos including ‘Top Tips on DNACPR communication’ to be made available in all Welsh health boards and trusts via Intranet, email and communications teams
- Promote videos and TalkCPR website as a resource area for healthcare professionals who want more information on details of DNACPR in Wales
- Make videos available to healthcare professionals via online and offline means, in order to share them with patients/carers and to supplement face-to-face discussions about this important topic
- Develop a strategic campaign to make videos widely accessible to general public, including on Youtube, Twitter, Facebook, charity websites and on television. Make videos smartphone compatible, but also develop resources that do not require internet access.

### PHASE 3- design

**Fig. 2:**
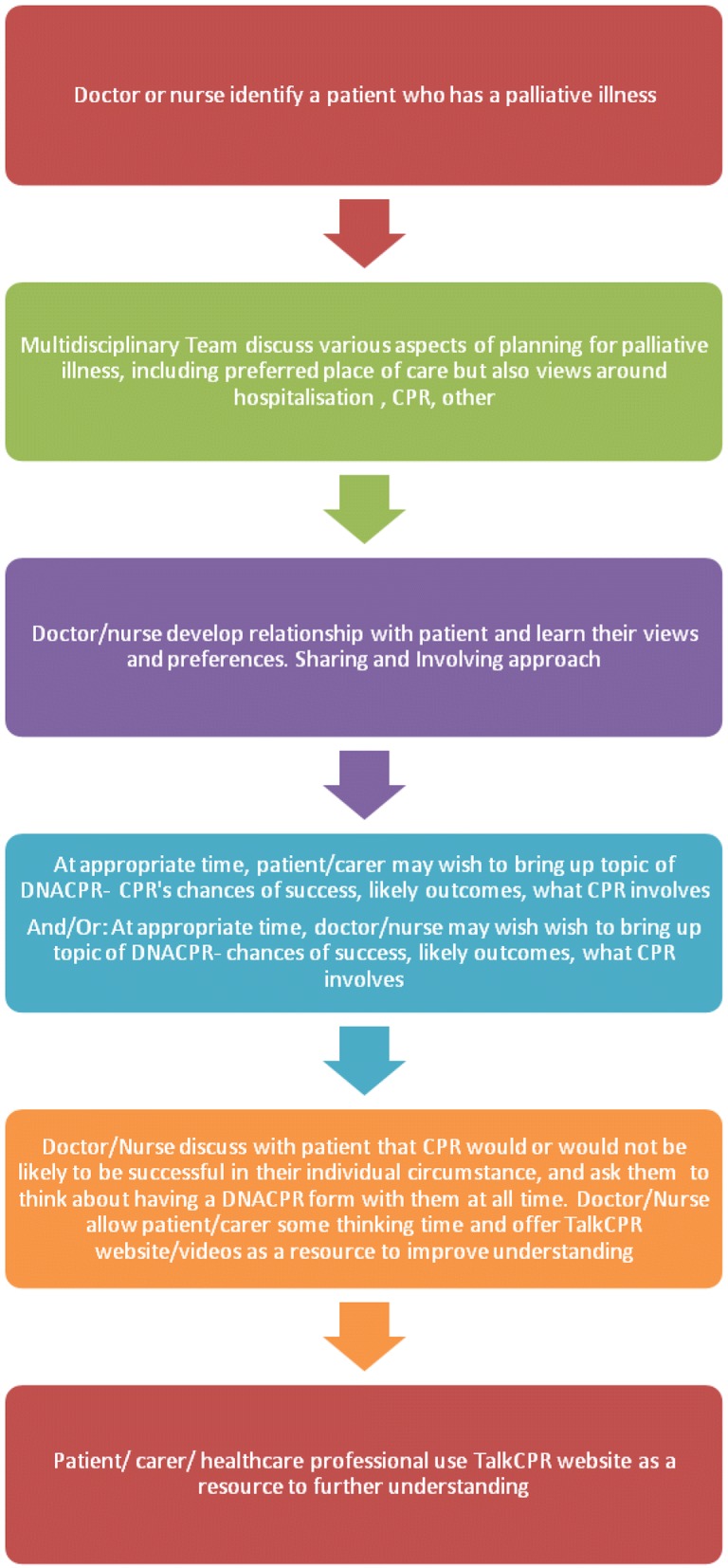
Process map of how DNACPR discussions in palliative care situations should be approached in Wales

Before creating the videos, the team also set out a process map on how DNACPR videos should be accessed and when (Fig. 2). Help to design the project around video production was obtained from the End Of Life Care coalition for Wales ‘Byw Nawr/Live Now’, Welsh Government, the End Of Life Care board for Wales who agreed funding for this project, and the Bevan Commission, who listed this as an exemplar project for Wales. Website and App design was co-ordinated by the Digital Legacy Association and the Welsh NHS IT services.

Framing what would trigger using this technology resource in the patient/carer journey, the following was agreed on by the project team:

- At appropriate time, patient/carer may wish to discuss appropriateness of potential future DNACPR decision- CPR’s chances of success, likely outcomes, what CPR involves.
- And/or: At appropriate time, doctor/nurse/healthcare practitioner may wish to bring up topic of DNACPR in a palliative care situation- including chances of success, likely outcomes, what CPR involves.

A healthcare practitioner (HCP) could discuss with a patient/carer that CPR would or would not have likelihood of success in a given situation, and explore the individual’s view on this topic. If appropriate, a suggestion could be made to write a DNACPR form and keep with patient/carer at all times, as per NHS Wales DNACPR policy. In addition, the healthcare professional should offer the patient/carer some thinking time before sharing their views, and offer TalkCPR website/videos as a resource to improve understanding and consent. This could be accessed via a webpage on patient’s/carer’s own computer device or on a video media pad left at house or by the hospital bed.

### PHASE 4- video production and website design

Over a period of 4 months, patients, carers, healthcare professionals and a video production company (Gingenious) created English language and Welsh language videos to inform anyone viewing these films what Cardiopulmonary Resuscitation (CPR) in life limiting and palliative illness involved. Scripting and production took 4 months in total, with a further post-production period of 2 months. Videos were hosted on the TalkCPR website (Additional file 1: Image 1). A Welsh language version of the TalkCPR website (Additional file 2: Image 2*)* was created http://talkcpr.cymru. Lightweight video media pads were produced, and had videos pre-loaded onto them, in order for healthcare professionals who wanted to bring videos to patients/carers in areas with less Wi-Fi or data access to still be able to do so. Videos were also produced for the hard of hearing (Additional file 3: Image 3), and an audio version for the visually impaired was also created.

### PHASE 5- evaluation strategy

Surveys were created to evaluate the videos. The aim was to establish whether videos were seen as a meaningful change and how acceptable and sensitive they were. Three sets of results are presented:

- A survey given to 25 nurses measuring pre- and post TalkCPR video viewing impact metrics (*n* = 25)
- A survey given to 15 junior doctors measuring pre- and post TalkCPR video-viewing impact metrics (*n* = 15)
- A summary of a hospital patient/carer focus group session (*n* = 14)
- A survey to evaluate TalkCPR video media pads with 100 healthcare professionals (*n* = 100)

**Fig. 3:**
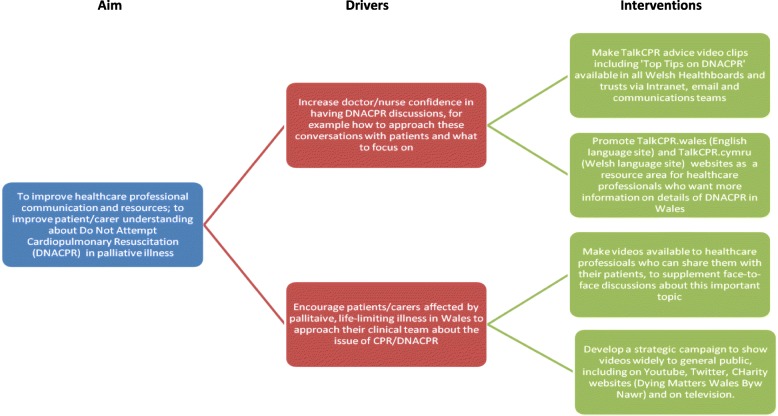
Survey Questions. Respondents filled in the survey twice, before and after viewing TalkCPR videos

The 25 nurses were participating in a palliative care update day, and came from many areas of hospital and community healthcare, including intensive care and district nursing. The hospital junior doctors were at that time job-rotating to Velindre NHS Trust. Survey questions for doctors and nurses can be viewed in Fig. 3.

Nurses and doctors filled in the questionnaire (baseline survey), then watched the TalkCPR videos and then filled in the questionnaire again (post intervention survey) on a separate sheet of paper, to measure if there had been any change in views and confidence after watching videos. They scored from 0 to 10 (0 = not ready at all, 10 = very ready).

We conducted a focus group session with the Velindre NHS Trust patient/carer liaison group. TalkCPR videos were presented to the Patient/Carer Liaison Group (PLG) at Velindre NHS Trust in December 2015. Fourteen members of the patient/carer group were present. Notes were taken during the meeting. Comments and suggestions were captured. Participants were asked a number of pre-prepared questions, for instance on the acceptability of the videos and whether they evaluated the videos as appropriate for viewers. Eight outcomes were eventually agreed on after discussion.

TalkCPR video media pads survey: A survey was completed by 100 healthcare professionals in NHS Wales (25 GPs, 25 junior doctors, 25 nurses, 25 hospital consultants) to look at usability of light weight video media pads, onto which the TalkCPR videos were pre-loaded and ready to view at the click of a button. A short film of the video catalogue being used can be viewed here: https://www.youtube.com/watch?v=5Zw9mD3K0pU.

A questionnaire was designed in order to evaluate the video media pad product amongst healthcare professionals. Input was received from a clinical psychologist with expertise in questionnaire design. From the questionnaire it was aimed to attain the following information:

1. The role of the person
2. How often they typically discuss DNACPR (0–3 / 3–6 / 6–10 / 10+ x per year)
3. Whether they thought the video booklet would facilitate DNACPR discussions (Yes/No)
4. How useful they thought patients/carers would find the video booklet (Yes/No)

Any nurse, junior doctor, consultant or General Practitioner working in Wales was entitled to take part and evaluate this video book product. Healthcare professionals were initially contacted via email, asking them to take part and were then met face-to-face, at which point they watched the videos on the video book, before finally filing in the survey.

Four sets of results were obtained from the following professional groups:

- Nurses (*n* = 25)
- Junior doctors (*n* = 25)
- Hospital consultants (*n* = 25)
- General Practitioners/General Practitioner trainees (*n* = 25)

## Results

### Nurses in TalkCPR pre- and post video evaluation surverys

**Fig. 4:**
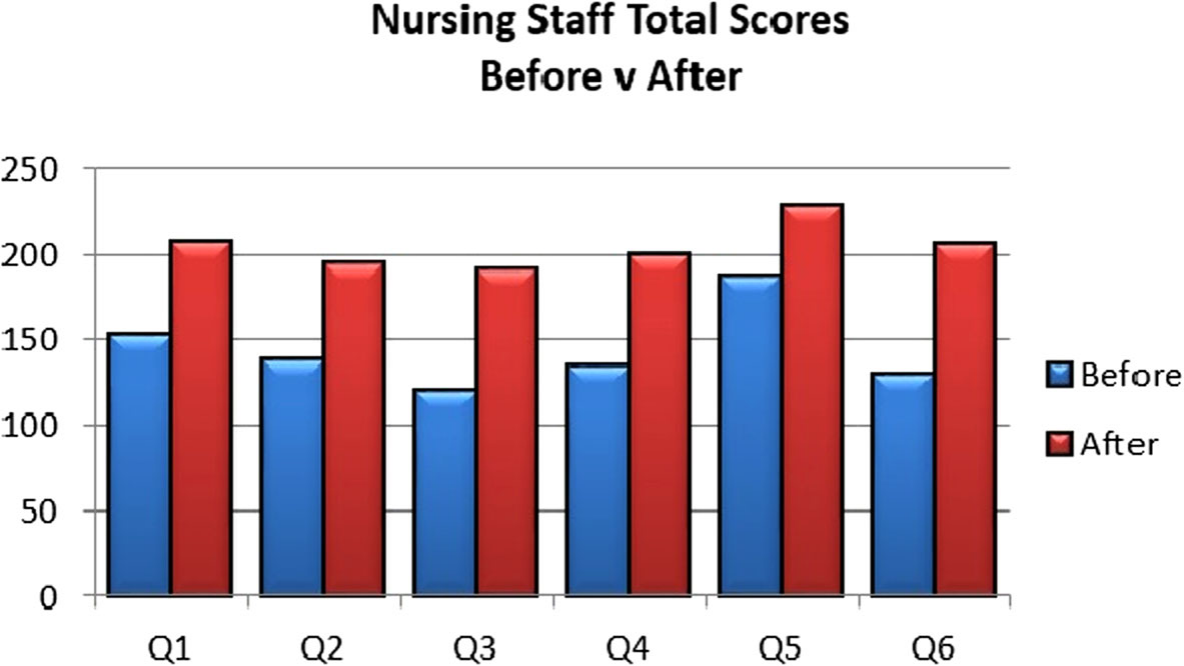
Nurses total confidence scores in survey to each Question (Q1-Q5), before and after watching videos

Average score across 25 general nursing participants through all questions was 6.12 out of 10 initially (0 = no confidence, 10 = maximum confidence), and increased to 8.28 out of 10 after watching the TalkCPR videos. A summary is provided in Fig. 4. This shows that despite initial (pre-video) lower confidence figures with regard to considering a video when broaching the topic of CPR and DNACPR, confidence levels subsequently increased once the nurse had viewed the TalkCPR videos.

### Doctors in TalkCPR pre- and post video evaluation

**Fig. 5:**
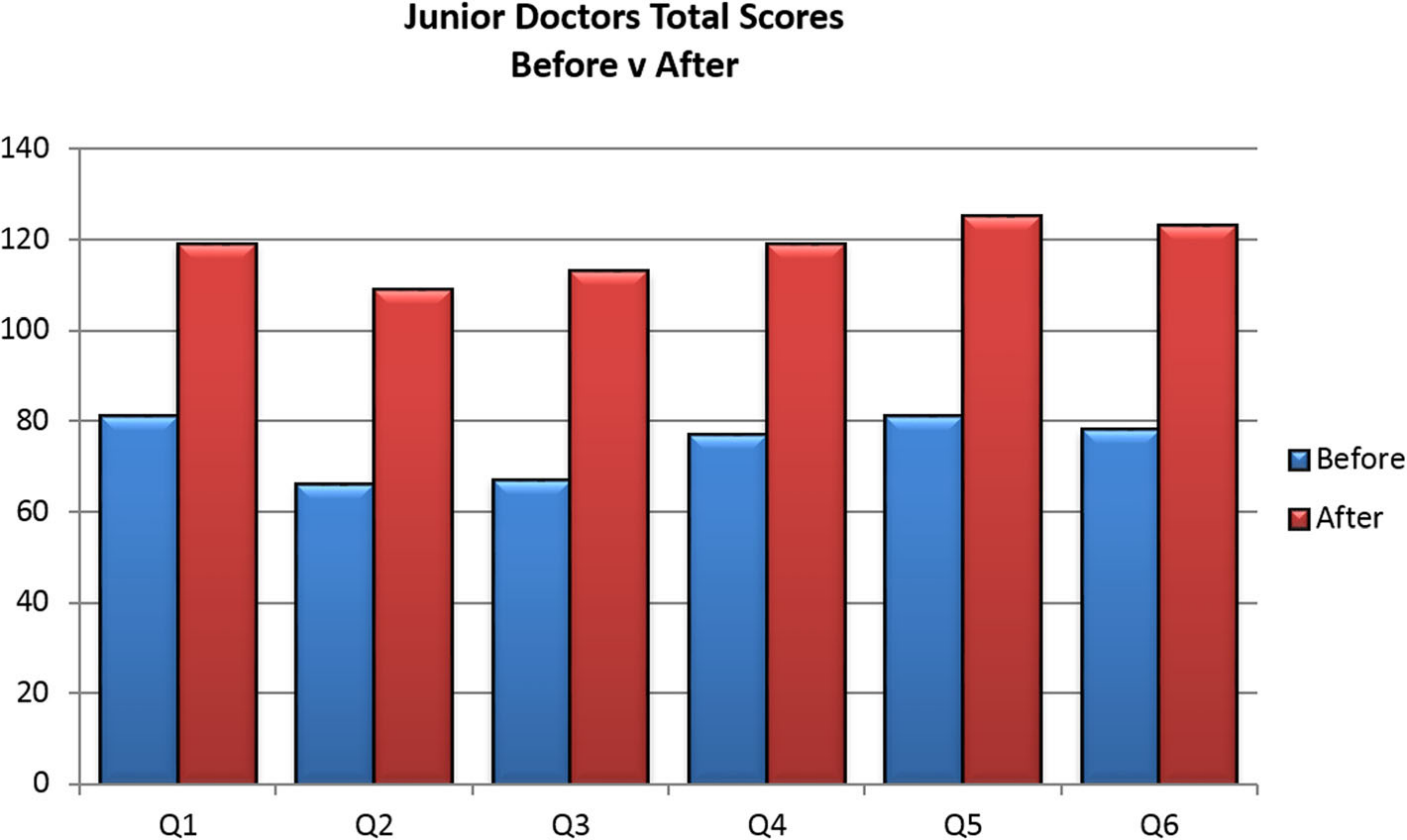
Doctors total confidence scores in survey to each Question (Q1-Q5), before and after watching videos

The same survey was given to junior doctors (*n* = 15) rotating through Velindre NHS Trust in Cardiff during 2015 and 2016 at the start of DNACPR teaching sessions, in induction week (Foundation Year 2- Core Training Year 2 level). They showed considerable increases in readiness to recommend videos to patients and proxy and they demonstrated increased confidence levels to show such a video, compared to pre-video survey. Figure 5 illustrates these findings.

### Patient liaison group (PLG) review of videos

Listed below are the outcomes of the PLG focus group meeting. These were agreed topics during the December meeting and resulted from round table discussion. In summary

- Outcome 1: PLG felt that videos were acceptable and not distasteful.
- Outcome 2: PLG took key educational messages from videos and interpreted them as was intended
- Outcome 3: PLG felt initial dissemination was appropriate and that videos should get wide-spread coverage
- Outcome 4: PLG agreed that wider remit (i.e. not merely in cancer settings) of video accessibility was highly appropriate
- Outcome 5: Black & white video was seen as highly informative and with potential to alter patients/carers preconceptions
- Outcome 6: Some PLG members felt that background music in videos was unnecessary and potentially distracting, others did not agree that this was a problem.
- Outcome 7: Mixed views on first (introductory) video showing a family in a waiting room, PLG felt that more detailed information on CPR/DNACPR could have been provided in this video- However acknowledged that this info was covered in other, longer video and the initial short video was aimed at generating interest in topic, suitable for social media channels as a ‘hook’.
- Outcome 8: Agreed that additional supporting information could be provided on TalkCPR website itself, for those who wanted more information after viewing first video.

A later suggestion from a patient liaison group member was to add subtitles to videos, and this was also fed back from a nurse who had tried to view videos from an NHS Health board computer that had no loudspeakers. Videos were re-edited and subtitles were added in response to this feedback.

### Survey regarding light weight video media pads

Frequency of DNACPR interactions per professional group in preceding year was recorded (*n* = 100; *n* = 25 per professional group; total of 4 professions). Table 1 shows the frequency of times each healthcare professional reported that they had discussed DNACPR in the previous 12 months.

**Table 1:** Frequency of DNACPR interactions per professional group in preceding year

	0-3 times	3-6 times	6-10 times	10+ times
Nurses	15	5	3	2
Junior doctors	9	3	4	9
Consultants	8	2	1	14
GP’s	7	10	4	4

**Fig. 6:**
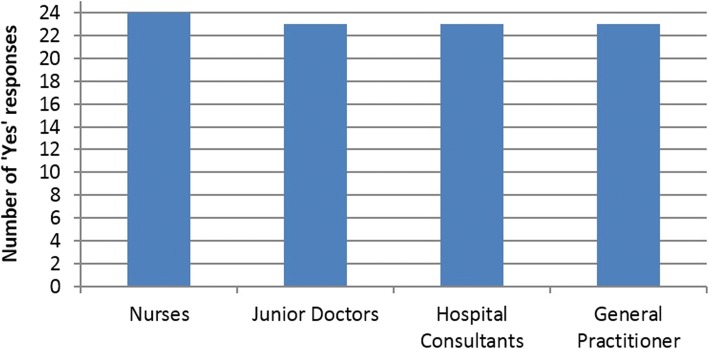
Number of ‘Yes’ respondents in survey per profession (n = 25 per profession) in answer to the Question: ‘Would this video media pad have been useful in facilitating your recent DNACPR discussions?’

**Fig. 7:**
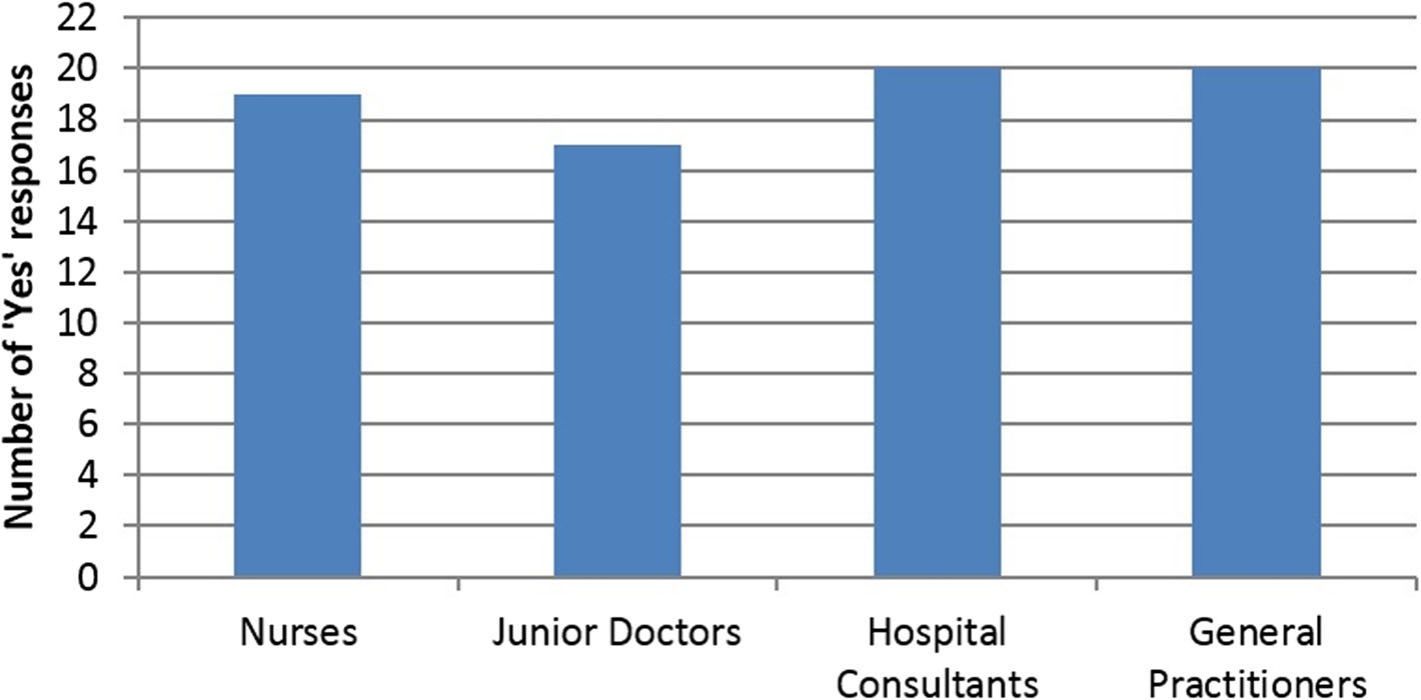
Number of Yes respondents in survey per profession (*n* = 25) in answer to question: ‘Do you think that patients would find the video media pad helpful after an initial face-to-face consult?’

Figures 6 and 7 are graphic representations of the results from answers in the questionnaire. For the survey question: ‘Would this video media pad have been useful in facilitating your recent DNACPR discussions?’, the number of ‘yes’ respondents per profession (*n* = 25 per profession) was recorded and is shown in Fig. 6.

The number of ‘Yes’ respondents per profession (*n* = 25) in answer to the survey question: ‘Do you think that patients would find the video media pad helpful after an initial face-to-face consult?’ is shown in Fig. 7.

The graphs demonstrate that the TalkCPR video media pads received a positive response across all professions.

The free text comments in surveys was evaluated and has been divided into healthcare professional categories below.

#### Nurses

Fourteen of the 25 nurses wrote additional comments on the video media pad surveys. Two of them commented that the devices were “*easy to use*”, whilst four nurses reported that in their opinion the devices would be “beneficial for relatives” after a discussion with the patient.

#### Junior doctors

Nine junior doctors provided additional comments on their surveys. Three wrote that if they were readily available on the wards that they would use them as a supplement and consider leaving them with the patient after the discussion as a platform for them to discuss DNACPR with their families.

Two wrote that they thought the videos would be valuable as a television advertisement or available in GP surgeries.

#### Hospital consultants

Twelve consultants shared their comments. One wrote that in their job as a consultant breast surgeon they would use the booklet to improve their skills. A Rheumatologist wrote that if the occasion arose, he/she would be likely to use the video booklet, however they added “*in clinical rheumatology the discussion is not had frequently*”. Two of the respondents were palliative care consultants; they agreed that the initiative was excellent and scored very low on the scale when asked how likely they would be to use them (with scores of 2 and 3), with one of them commenting that “DNACPR conversations are the bread and butter of palliative care” and therefore they would be unlikely to use such additional resources.

#### General practitioners

One GP wrote that “for some patients such as technological material would prove confusing and may enhance their feeling of alienation and confusion” however other than this the comments were only positive.

Three GP’s wrote that they would be a very useful tool to keep in their bag for house calls, with one adding “*often I arrange to revisit patients a few days after the initial discussion; it would be useful to leave something for the patient to watch between my visits to help them make an informed decision. I think this resource would be well placed in primary care”.*

Another wrote “*it would help to reinforce the topics discussed during the consultation as a few patients continue to have unanswered questions and fears despite my best efforts at reassurance and explanation. I feel the video booklet would be helpful in addressing such issues”.*

## Discussion

Videos are now available in each Welsh health board on intranet sites, and centrally on the http://talkcpr.wales website (Additional files 1, 2, 3: Images 1, 2, 3). The project has received considerable international attention, in part due to the aforementioned Guardian article, but also due to media attention from a BMJ Supportive and Palliative care blog, which was read out initially by the singer Jarvis Cocker and subsequently by the actor Benedict Cumberbatch at literary events [9, 10].

A major direction for Welsh Government in future, and the healthcare profession more generally, is future and advance care planning using shared decision tools. Videos, apps and websites are one way of facilitating this and technology may replace the traditional method of handing out numerous paper patient information leaflets.

TalkCPR videos, website and media pads have been quality assessed and evaluated by a patient/carer group, junior doctors, GPs, consultants and nurses, and were found to have a significant impact on confidence to address the topic of DNACPR cogently and confidently in each group.

The daughter of the patient at the centre of the Tracey versus Cambridge court case has supplied the TalkCPR project and website with the following testimonial:

*“The TALKCPR website is the first widely available resource I have come across. The bite size videos are easily accessible and provide a fair bit of information, but their greatest value to me is that even if they don’t answer all the questions you have, they may provide the information needed to start the much needed conversation about CPR and DNACPR and end of life choices with your doctor. The way the menu links straight to the FAQs page is greta, and I particularly like the 50 second video overview of the site – is it a TV ad? If not, it should be!*” - Kate Masters, daughter of Janet Tracey

**Fig. 8:**
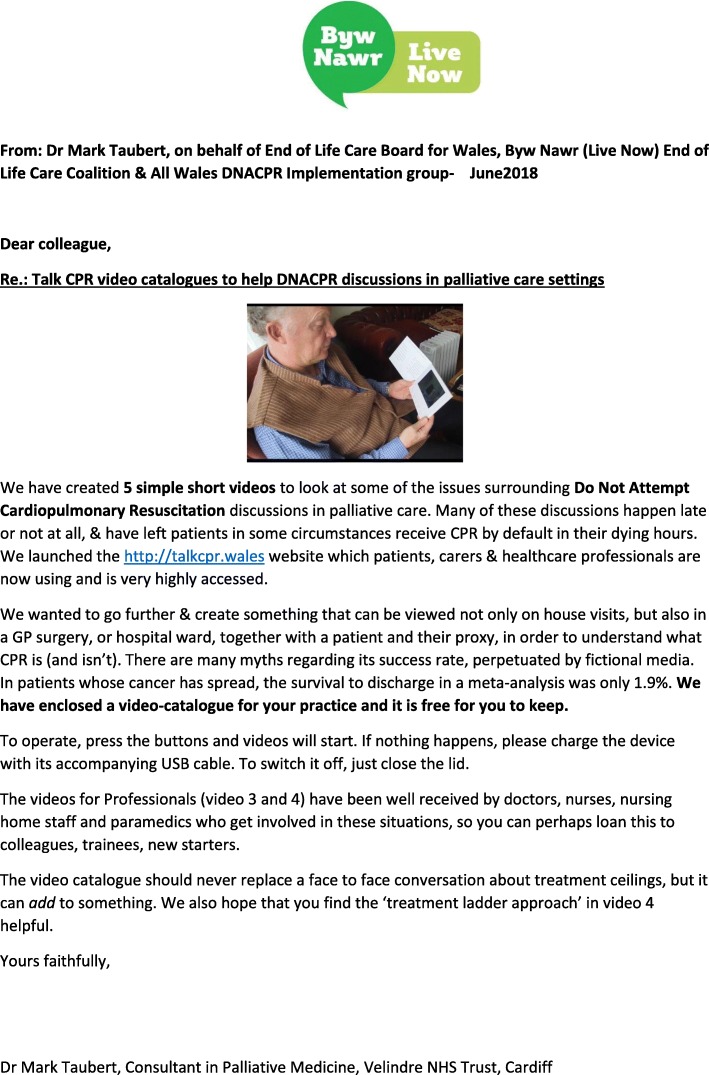
Letter to GPs that accompanied the TalkCPR video media pads. Each practice in Wales received one TalkCPR video media pad

Limitations and challenges during the running of the project were documented and discussed. They included the difficulties of creating video resources for various health boards with very different IT systems (some health boards did not have the up-to-date Windows software to access videos, for instance) and issues such as sound and loudspeaker access on computers in different healthcare settings. There were reports of variability in access to internet and Wi-Fi in different parts of Wales, with urban settings more likely able to gain access and bring the videos to the patient bedside or home. The creation of small lightweight video media pads that play all TalkCPR videos at the press of a button, without need for wi-fi or data access, enabled access for more remote areas. Video media pads have been sent out to all GP practices and clusters in Wales with a covering letter (Fig. 8).

## Conclusions

This is a new approach to tackling the sensitive issue of DNACPR discussions, using multiple technological media including websites, videos and media pads aimed at patients, carers and healthcare professionals alike.

In order to have discussions about CPR/DNACPR that are *both* routine and meaningful, a new direction of travel is required. Discussions can be made more *routine* by having them at predictable junctures (perhaps in the community and ideally with known practitioners) and increasing patients’ education about the need for such discussions. These same interventions may also render discussions more *meaningful*.

The technology used, i.e. videos, website and video media pads received very positive comments. The high proportion of junior doctors and consultants who reported discussing DNACPR more than ten times in the last 12 months (shown in Table 1) could reflect the fact that these discussions are often left until patients have acutely deteriorated and are admitted to hospital. This highlights the need for additional resources to be applied to general practice to ensure that these discussions happen earlier under more favourable circumstances for the patient and their loved ones. Our survey on TalkCPR video media pads also indicated a high demand for TalkCPR media pads from General Practitioners filling in the survey.

Beneficiaries from technology interventions like TalkCPR will also include those patients who do not wish to have life-saving treatments attempted, but have never been given an explicit opportunity to say so. This is prudent healthcare at its most patient-empowering and forward planning. We have not attempted to outline the cost of CPR events, including those that fail, but there is existing literature that gives evidence to that effect.

The campaign has been widely publicised on social media, for instance via the hashtag #TalkCPR on Twitter. After less than 6 months of being ‘live’ the site had reached nearly 100,000 hits. Comments on social media have been extremely positive and encouraging and no one has stated that the videos ae inappropriate or insensitive. By addressing the public directly with the TalkCPR videos and resource website, and also using videos as a learning resource for healthcare professionals, we hope to have created one of the steps towards an open, transparent process to capture people’s views on this sensitive topic. The project has won the NHS Wales Award 2016 in the Improving Quality Together using IQT Methodology category. It won a runner-up prize at the BMJ Awards in 2017. A collaboration with Falmouth University School of Graphic Design has meant that art students as part of the Moth art project have helped with innovative visual and video aids to encourage DNACPR discussions. Based on the survey we conducted on 100 Healthcare practitioners on the Talk CPR video media pads, we sent one pad out with a covering letter (see Fig. 8) to each GP practice and GP cluster in Wales (*n* = 427). It is hoped that this will provide an impetus for open and honest anticipatory face to face conversations on future ceilings of treatment in people with serious palliative illness.

### Availability and requirements

Project name: TalkCPR.

Project home page: http://talkcpr.wales

Programming language: PHP, HTML, centOS.

Requirements: Open access via web. Smartphone and tablet compatible.

License: NHS Wales. No third party/ongoing licenses or license costs.

Restrictions: None.

## Additional files

Additional file 1: **Image 1.** TalkCPR website- English language version. (PNG 479 kb)

Additional file 2: **Image 2.** TalkCPR- Siarad am CPR – Welsh language version. (PNG 465 kb)

Additional file 3: **Image 3.** Sign language video. (PNG 611 kb)

## Abbreviations

ACP: Advance Care Planning
CPR: Cardiopulmonary resuscitation
DNACPR: Do Not Attempt Cardiopulmonary Resuscitation
DNR: Do not resuscitate
GP: General practitioner
HCP: Healthcare practitioner
IT: Information Technology
NHS: National Health Service (United Kingdom)
NICE: National Centre for Health and Care Excellence
PLG: Patient/Carer Liaison Group at Velindre NHS Trust in Cardiff, UK
