# Supplementary figures and images for: Talk CPR - a technology project to improve communication in do not attempt cardiopulmonary resuscitation decisions in palliative illness
Source: BMC Palliat Care. 2018 Oct 19;17:118. doi: 10.1186/s12904-018-0370-9 (PMC6195698; doi:10.1186/s12904-018-0370-9)

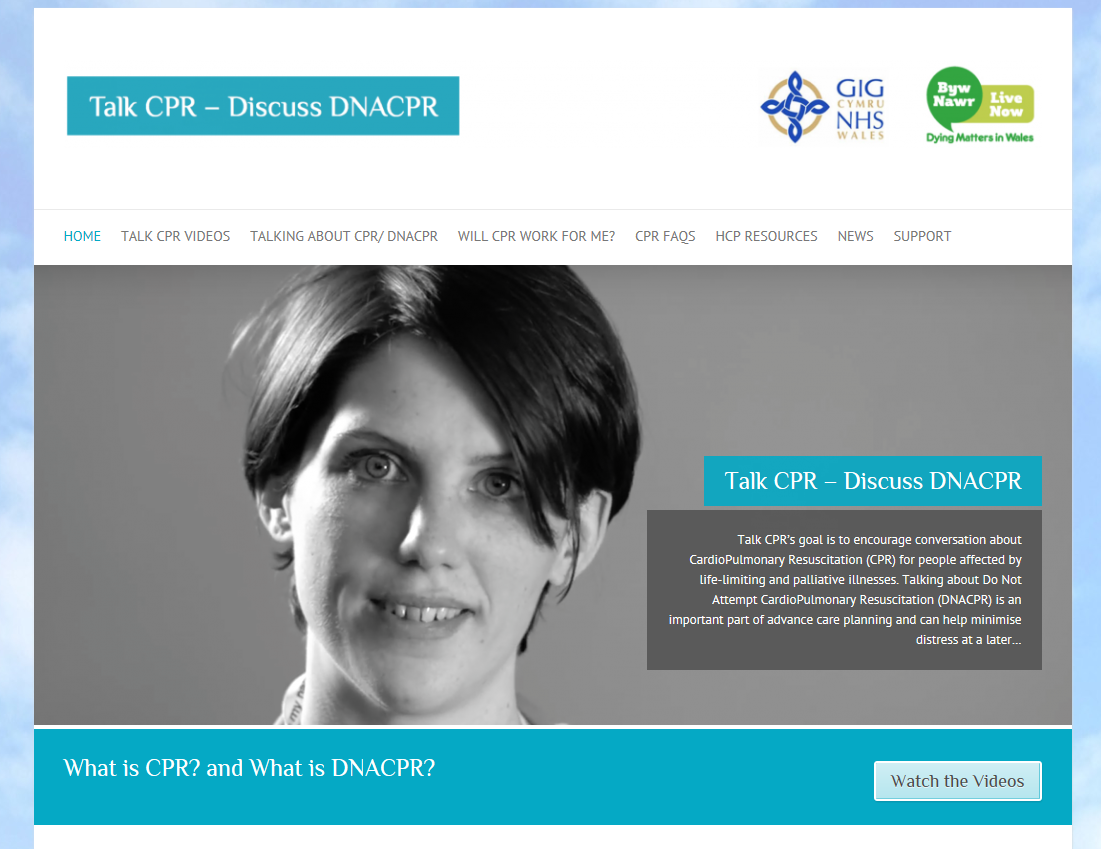

Supplement: Supplementary file 1 — Image 1. TalkCPR website- English language version. (PNG 479 kb) [file 12904_2018_370_MOESM1_ESM.png]

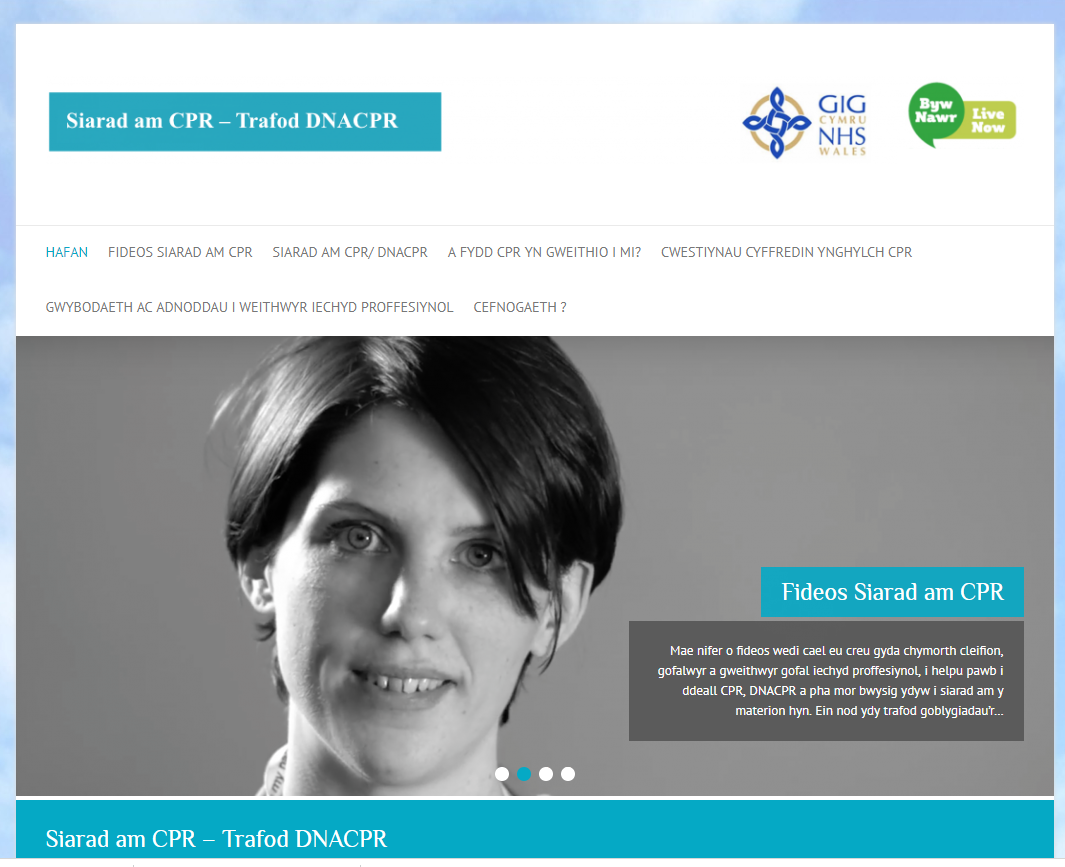

Supplement: Supplementary file 2 — Image 2. TalkCPR- Siarad am CPR – Welsh language version. (PNG 465 kb) [file 12904_2018_370_MOESM2_ESM.png]

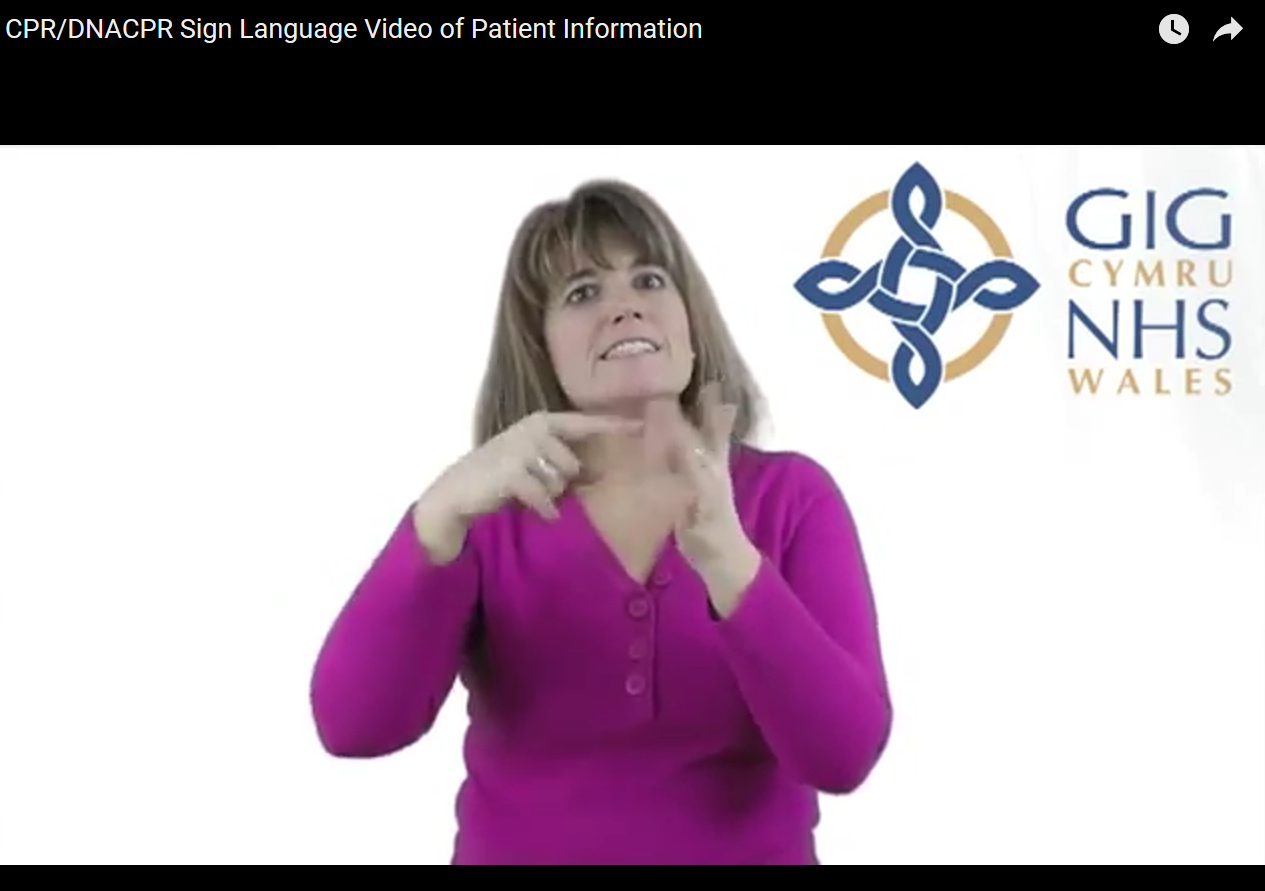

Supplement: Supplementary file 3 — Image 3. Sign language video. (PNG 611 kb) [file 12904_2018_370_MOESM3_ESM.png]
